# Association of household socioeconomic status, neighborhood support system and adherence to dietary recommendation among persons with T2DM, a facility-based cross-sectional study in Ghana

**DOI:** 10.1186/s12889-021-10963-x

**Published:** 2021-05-13

**Authors:** Be-Ikuu Dominic Doglikuu, Abdulai Abubakari, Mehdi Yaseri, Elham Shakibazadeh, Abolghassem Djazayery, Khadijeh Mirzaei

**Affiliations:** 1 International Campus, Department of Community Nutrition, School of Nutritional Sciences and Dietetics, Tehran University of Medical Sciences (TUMS), Tehran, Iran; 2 Ministry of Health, Nursing and Midwifery Training College, Twifo Praso, Central Region Ghana; 3 Department of Nutritional Sciences, School of Allied Health Sciences, University of Development Studies (UDS), Tamale, Ghana; 4 Department of Epidemiology and Biostatistics, School of Public Health, Tehran University of Medical Sciences, Tehran, Iran; 5 Department of Health Education and Promotion, School of Public Health, Tehran University of Medical Sciences, Tehran, Iran; 6 Department of Community Nutrition, School of Nutritional Sciences and Dietetics, Tehran University of Medical Sciences (TUMS), Tehran, Iran

**Keywords:** Diabetes mellitus, Socioeconomic-status, Neighborhood support, Adherence, Dietary recommended, Ghana

## Abstract

**Background:**

Dietary recommendation help persons with diabetes adopt to healthy eating habits to achieve optimal glycemic control. Socioeconomic-status and neighborhood support system can influence adherence to dietary recommendation. The purpose of our study is to assess the association of household-socioeconomic status and neighborhood-support system with adherence to dietary recommendation among persons with type 2 diabetes mellitus (T2DM).

**Methods:**

Facility-based cross-sectional-survey was conducted in Brong Ahafo region, Ghana. Six hospitals were randomly selected and 530 individuals with T2DM consecutively recruited from the selected hospitals for the study. Structured-questionnaires were used to collect socio-demographic variables. Adherence to dietary-recommendation was the outcome-variable, and was assessed using perceived dietary-adherence questionnaire.

**Results:**

Age (years) (*P*-value = 0.005), Physical-Activity level (*P*-value = 0.024) Receive-moderate Social-Support (*P*-value = 0.004) and High-Socioeconomic status (*P*-value = 0.046) were significantly correlated with adherence to dietary-recommendation. Age (years) regression coefficient (β) -0.089, 95%CI (− 0.12, − 0.001), Being married β0.103, 95%CI (0.002, 0.02), moderate and low-social support system β 0.309, 95%CI (0.17, 0.38) and β-0.192, 95%CI (− 0.26, − 0.06) respectively, and high-socioeconomic status β 0.197, 95%CI (0.06, 0.25) were significantly associated with adherence to dietary-recommendation.

**Conclusion:**

Social-support system and socioeconomic-status could be associated with adherence to dietary-recommendation. Therefore, health workers should consider patients’ social support system and socioeconomic status as modifiable factors for optimum adherence.

## Introduction (Background)

Diabetes Mellitus (DM) is a public health problem of which dietary recommendations forms integral part in its management [1]. Dietary Recommendation for diabetes management help persons with DM adopt healthy eating habits to achieve optimal glycemic control [2]. These dietary recommendations often focus on counseling patients with DM to control glycaemia by matching dietary carbohydrate intakes with medications [3]. Despite this, DM still contributes significant public health threat to individuals and society [4]. Sustaining complex array of lifestyle modifications and self-care behavior practices such as medications intake, adherence to dietary recommendations, regular physical activities and monitoring of blood glucose levels [5, 6] are important factors in DM management. However, adherence to dietary recommendations are considered corner stone [7]. Appropriate dietary recommendations for diabetes management emphasizes the intake of: Diets rich in whole grains, Fruits, Vegetables, Legumes and Nuts [8]. It also emphasizes the intake of Moderate alcohol; Low refined grains, Low red and processed meats, and Low sugar-sweetened beverages [8]. Furthermore, it emphasizes on the intake of: Less fat, Less sodium, More fiber, and More foods such as fish and soy products that have health-promoting properties [8]. Research shows that improving adherence to dietary recommendations helps persons with DM reduce glycosylated hemoglobin (HbA1c) level by 1 to 2% absolute term with the greatest effect felt at initial stages of diabetes [9]. Despite these remarkable effects of dietary recommendation in diabetes management, variation in financial ability and social support (both from family and friends) could cause inappropriate adherence. Financial status and social support systems are powerful variables for therapy adherence in stressful situations. Studies show that increasing appropriate social support systems can act directly to encourage individuals with chronic non-communicable diseases like DM to adopt healthier behaviors such as reducing unhealthy dietary intakes, participate in physical activities, or give up smoking which are risk factors for disease onset and progress [10]. Although it is known that socioeconomic status and social support systems are strong predictors for therapy adherence in diabetes management, little is known about how these variables are associated with adherence to dietary recommendations among persons with type 2 diabetes mellitus (T2DM) in Ghana. Dieticians in Ghana often offered T2DM patients individualized dietary counseling based on the recommended dietary guidelines for diabetes management. However, it is unclear whether socioeconomic status and social support system play significant roles for adherence to these individualized nutrition care plans among patients. Ghanaian population lives in heterogeneous communities with diverse socioeconomic status cultural practices, ethnic grouping and educational level. Due to this there are variations in cultural practices, social support networks and income levels among the peoples. Yet no study has been conducted to explore the association of these variables for adherence to dietary recommendations among persons with diabetes.

## Method

The aim of our study is to investigate how household socioeconomic status and neighborhood support system are associated with adherence to dietary recommendation among persons with T2DM in Ghana. Hospital based cross-sectional survey was conducted among 530 persons living with DM in Brong Ahafo Region (BAR), Ghana. Single population proportion formula ($n=\frac{{\mathrm{Z}}^2\mathrm{P}\left(1-\mathrm{P}\right)}{e^2}$) was used to determine the sample size for this study. The letter ‘n’ in the formula denotes the study sample size, ‘Z’ denotes normal standard distribution of 1.96 for 95% confidence interval, ‘P’ is the true population proportion of adherence to dietary recommendation among DM persons in the study area (Brong Ahafo region) and ‘e’ is standard error (5%). Previous study in Brong Ahafo region Ghana, reported that prevalence of adherence to dietary recommendation is 68.5% [11]. Substituting these values in the equation above, the sample size n was calculated as $n=\frac{1.96^2\ast 0.685\left(1-0.685\right)}{0.05^2}$ = 332. However, for the event of non-response and registration error, a contingency sample of 60% was considered in the sampling, therefore the final sample was increased to 0.6 ∗ 332 = 531.2 ≈ 532.

Individuals 18 years and above who were diagnosed with T2DM by physicians, using the American diabetes association (ADA) diagnostic and classification guideline 2011 [12], and counseled to follow recommended dietary guidelines for at least 3 months and over were recruited. Participants’ 70 years and above who could not answer interview questions, intellectually deficient, and severely ill were excluded. Pregnant and lactating mothers were also excluded. Simple random sampling was used to select 6 hospitals, and the eligible participants consecutively recruited using systematic random sampling.

### Ethical approval

The study protocol was approved by Ghana Health Service Ethics Review Committee (GHS-ERC008/08/18) and Tehran University of Medical Sciences Ethics Review board (IR.TUMS.VCR.REC.1397.409). Each participant was requested to sign an informed consent form before participating. This research project was performed in accordance with the Declaration of Helsinki.

#### Assessing patients demographic characteristics anthropometry measurements and clinical parameters

Age, diabetes-duration, medications intakes and other demographic characteristics were assessed using structured questionnaires. Weight and height were measured and recorded to the nearest 0.5 kg and 0.5 m using adult weighing scale and stadiometer respectively. These measurements were taken while participants were in light clothes without shoes, and were in standing position. Body mass index (BMI, kg/m2) was calculated by dividing weight in kilograms with height in meters square. Systolic and diastolic blood pressures were measured using manual sphygmomanometer and stethoscope, and the reading recorded to the nearest 0.5 mmHg after participants were allowed to relax for 5 or more minutes.

#### Assessing socioeconomic status

We assessed participants’ socioeconomic status using composite wealth index. This proxy indicator was used because participants were unwilling to tell us their disposable household income they earn through sales and salaries per month. Using this method, we asked participants to name items and properties they possess and use in their homes including fixed assets like land and building, and movable assets like vehicles. We then used principal component analysis (PCA) to extract participants’ socioeconomic status from this wealth index. The extracted socioeconomic status was categorized into three quintiles: - poorest, middle and richest quintiles to represent participants’ socioeconomic status. After the extraction, the percentage of total variance explained by the three factors was 35.6%.

#### Neighborhood support system

We assess neighborhood support system by using structured questionnaire. Participants were asked to self-report on a continue scale, how frequent they received support in the form of materials gifts, cash, in kind or volunteerism from friends, relatives, love ones, or from religious organizations like churches,mosques, or from cooperate institutions in their societies. Participants who reported “very frequently” were classified to have high social support, those who reported “frequently” were said to have moderate social support, and those who reported “less frequently” were said to have low social support system.

### Assessing participants’ alcohol intake

WHO 10-items alcohol use disorder identification scale (AUDITs-10) [13] was also used to assess Participants’ alcohol intake level. Participants’ were asked to respond to the 10 points in AUDITs scale, ranging from *‘How often do you have drink containing Alcohol?* to *“Has a relative, friend, doctor, or other health care worker ever been concerned about your drinking and suggested that you cut it down?’* The responses obtained were summed up to form participants’ total alcohol intake status. ‘The responses to these questions were in likert’s scale of 1 = (Never), 2 = (2–4 times a month) 3 = (2–3 times a week), 4 = (4 or more times a week). Based on the scale category, patients who report ‘Never’ to all the item on the scale were said to have no alcohol intake history, those who reported intake of ‘2-4 times a month’ were said to have low alcohol intake history while those reported intake of ‘4 or more times a week’ were said to high alcohol intake history. These questionnaires were pretested among 20 participants (chronbach alpha of 0.55).

### Assessing participants’ smoking status

Fagerström 6-iterms nicotine dependency test scale was used to assess participants smocking status [14]. Participants were asked to respond to 6-iterms in the Fagerström nicotine dependency test scale, *ranging from ‘How soon after you wake up from bed do you smoke your first cigarette?’ to ‘Do you smoke even if you are so ill that you are in bed most of the day?’* Responses to these questionnaires ranging from 0 to 3,. Participants who obtained a sum of 0 for these questionnaires were said to have no smoking history. Those who obtaned a sum of 1 were said to have low smoking history and those who obtained a sum of 2 and above were said to have high smoking history. These questionnaires too were pretested among 20 people (chronbach alpha of 0.55).

#### Assessing physical activity levels

WHO recommendations on physical activity for health was used in analyzing Participants’ physical activity (PA) level in our study; in calculating the physical activity level of participants in our study, the total time spent in physical activity during a typical week and the intensity of the physical activity were taken into account. Throughout a week, including activity for work, during transport and leisure time, WHO recommends that adults should do at least 150 min of moderate-intensity physical activity OR 75 min of vigorous-intensity physical activity OR an equivalent combination of moderate- and vigorous-intensity physical activity achieving at least 600 MET-minutes. In work related physical activity, participant who reported moderate work is scored Moderate MET value = 4.0; those who reported vigorous work were scored Vigorous MET value = 8.0. In transport physical activity, participants who reported Cycling and walking were given MET value = 4.0. Furthermore participants who engaged in recreational physical activities were given Moderate MET value = 4.0 if they reported moderate recreational activities and Vigorous MET value = 8.0 if they reported vigorous recreational physical activities. The MET values of each physical activity level was multiply with the number of hours and number of days spent in doing that particular physical activity level. The results obtained from the participants were grouped with reference to WHO physical activity cut of points (Not meet recommendations if Total Physical Activity MET minutes per week are < 600) and (Meet recommendation if Total Physical Activity MET minutes per week are ≥600) [15].

#### Assessing adherence to recommended dietary guidelines

Perceived Dietary Adherence Questionnaires (PDAQ) for Persons living with T2DM was used to assess adherence to dietary recommendation [16]. These questionnaires consist of nine items and seven point likert’s scale designed to information about adherence to recommended dietary guidelines from  patients. These seven point likert’s scale questionnaires have a range between 0 and 7. Zero point mean non-adherence to the PDAQ, and 7 point means highest adherence. Participants’ responses from the nine items were summed up to form participants’ total adherence to dietary recommendation score. These questionnaires were pretested among 20 participants (chronbach alpha of 0.95).

#### Statistical analysis

IBM SPSS version 22.0 (SPSS, Chicago, IL, USA) was used in all data analysis. Data normal distributions were checked with Kolmogorov-Smirnov test. Descriptive statistics were used to describe participants’ demographic characteristics, while Pearson correlation used to test the correlation of these variables with socioeconomic status and adherence to dietary recommendation. Finally multiple linear regression models were used to assess the association of household socioeconomic status and neighborhood support system for adherence to dietary recommendation. Multiple linear regression model looks at the association between predictor variables on one dependent variable in an Eq. Y = a + b_1_X_1_ + b_2_X_2_ + _b3_X_3_ + b_4_X_4_ + b_5_X_5_ + b_k_X_k_ + e, where ‘a’ is the regression constant, b is the regression coefficient, X_1_ … …. X_k_ are the independent variables and ‘e’ is the variance of the population mean distributions. The assumption for using this statistic in our study is that our dependent variable (Adherence to dietary recommendation) is normally distributed and has equal variance around the mean. The independent variables also have linear relationships with no multicollinearity. Furthermore, our sample size is fairly large and could be said to have fair representation of the larger population. In our analysis, we set all variables significant at 0.050 alpha levels.

## Results

Participants’ anthropometry and general characteristics are presented in Table 1. Mean (SD) of total adherence to recommended dietary guideline was 32.56(9.61). Mean (SD) of age (years), and BMI (Kg/m^2^) were 58.10(9.70) and 23.14(2.92) respectively. Majority of participants (70.9%) were females; married (64.2%); and live in small towns and villages (76.2%). More than 38% of participants have no formal education; 1.9% has education up to polytechnic; 2.5% have it up to university and the rest have other form of education.

**Table 1:** Participants anthropometry and general characteristics

Variable	Means (SD)	N (%)
Adherence to dietary recommendation	32.56 (9.61)	
Age (years)	58.10 (9.70)	
BMI (Kg/m^2^)	23.14 (2.92)	
Duration lived with diabetes (years)	4.90 (5.40)	
**Sex**		
Male		154 (29.1)
Female		376 (70.9)
**Marital status**		
Married		340 (64.2)
Single		20 (3.8)
Widow		107 (20.2)
Divorce		63 (11.9)
**Place of Residence**		
Village		39 (7.4)
Town		404 (76.2)
City		87 (16.4)
**Educational Level**		
No education		202 (38.1)
Primary		85 (16.0)
Junior High		132 (24.9)
Senior High		67 (12.6)
Training College		21 (4.0)
Polytechnic		10 (1.9)
University		13 (2.5)

*Means (SD)* mean (standard deviation), *N (%)* number (percentage)

There were significant correlation between age (*p*-value = 0.005), Alcohol intake (*p*-value = 0.024), education level (*p*-value = 0.010), diabetes duration (*p*-value = 0.013), high socioeconomic status (*p*-value = 0.046), and receive moderate social support system (*p*-value = 0.004) with adherence to recommended dietary guidelines, Table 2.

**Table 2:** Correlation of participants’ demographic characteristics with adherence to dietary guideline

Variables	Correlation coefficient (r)	***P***-value
Age (years)	**0.159**	**0.005**
Smoking status	−0.070	0.107
Alcohol intake status	**0.098**	**0.024**
Physical Activity level	**0.098**	**0.024**
Education level	**0.142**	**0.010**
Diabetes duration (years)	**0.137**	**0.013**
High Socioeconomic status	**0.104**	**0.046**
Moderate Socioeconomic status	0.089	0.074
Low Socioeconomic status	0.035	0.284
Receive low social support	0.081	0.096
Receive moderate Social Support	**−0.164**	**0.004**
Receive high Social Support	0.075	0.111

### Association of participants’ socioeconomic status and neighborhood support system for adherence to dietary recommendation

The association of household socioeconomic status and neighborhood support system for adherence with dietary recommendations is presented in Table 3. After adjusting for possible confounding factors (medication intake and BMI), Age (years) was statistically significant for adherence to dietary recommendation ([Standardized regression coefficient (*β*) -0.089, 95% confidence interval (− 0.12,-0.001); *P*-value = 0.045); Marriage was statistically significant for adherence to dietary recommendation (*β* 0.103, 95%CI: (0.002, 0.02); *P*-value = 0.018) whereas Received moderate social support (*β* − 3.185, 95%CI: (− 0.26, − 0.06); *P*-value< 0.001) and low social support (*β* 5.097, 95%CI: (0.17, 0.38); *P*-value = 0.002) were statistically significant for adherence to dietary recommendation. Finally, high socioeconomic status was statistically significant for adherence to dietary recommendation (*β* 3.123, 95%CI: (0.06, 0.25); *P*-value = 0.002) and moderate socioeconomic status (*β* 2.080, 95%CI: (0.01, 0.25); *P*-value = 0.039).

**Table 3:** Association of participants’ socioeconomic status and neighborhood support system for adherence to dietary recommendation

Variables	Unstandardized regression coefficient(β)	Standard error	Standardized regression coefficient(***β)***	t	***P***-value	95%CI
Age (years)	**−0.058**	**0.029**	**−0.089**	**−2.009**	**0.045**	**(−0.12, − 0.001)**
Sex	0.117	0.080	0.065	1.463	0.144	(−0.04, 0.27)
Smoking	−0.203	0.108	−0.090	−1.880	0.061	**(**−.414, .009**)**
Alcohol intake	0.168	0.086	0.094	1.945	0.052	**(**−.002, .338**)**
Physical activity level	−0.027	0.048	−0.027	− 0.565	0.572	**(**− 0.12, 0.07**)**
Married	**0.009**	**0.004**	**0.103**	**2.378**	**0.018**	**(0.002, 0.02)**
Place of Residence (urban)	0.018	0.012	0.068	1.571	0.117	(−0.01, 0.04)
Diabetes. Duration (years)	0.001	0.004	0.013	1.399	0.162	(−0.01, 0.04)
Education level	**0.151**	**0.074**	**0.088**	**2.043**	**0.042**	**(0.01, 0.29)**
High Social Support	0.013	0.051	0.015	0.247	0.805	(−0.09, 0.11)
Moderate Social Support	**0.271**	**0.053**	**0.309**	**5.097**	**0.000**	**(0.17, 0.38)**
Receive low social support	**−0.162**	**0.051**	**−0.192**	**−3.185**	**0.002**	**(−0.26, − 0.06)**
Socioeconomic High	**0.154**	**0.049**	**0.197**	**3.123**	**0.002**	**(0.06, 0.25)**
Socioeconomic Moderate	**0.126**	**0.061**	**0.128**	**2.080**	**0.039**	**(0.01, 0.25)**
Socioeconomic Low	0.034	0.049	0.041	0.689	0.491	(−0.06, 0.13)

The models were adjusted for medication intake, smoking, alcohol intake, physical activity and BMI

## Discussion

Socioeconomic status and neighborhood support system are strong predictors for therapy adherence in diseases management [17]. Our first objective was to investigate whether participants’ demographic characteristics were correlated with adherence to dietary recommendation. The second was to access whether household socioeconomic status and neighborhood support system were statistically significant for adherence to dietary recommendation using the multiple linear regression models. At the end of our analysis we realized that Age, Alcohol intake, education level, diabetes duration, high socioeconomic status and receive moderate social support were statistically correlated with adherence to dietary recommendation. We also noticed that decreasing age (years) was statistically significant for adherence to dietary recommendation, while being married, and increasing education level were statistically significant for adherence to dietary recommendation. Furthermore reducing moderate social support system was statistically significant for adherence to dietary recommendation while high socioeconomic status was statistically significant for adherence to recommended dietary recommendation.

As it can be recalled from the demographic analysis in Table 1, we realized that majority of our participants are female, married, have no formal education, and lived in small towns and villages. In Ghana there are disparities in wealth and resources distributions across gender, age, location and educational achievement, of which, the less educated, rural dwellers, the aged and women are vulnerable. As wealth or income distributions and educational achievements play critical role in healthcare seeking behaviors and services utilization, rural dwellers, the less educated, aged, women and people at the lower quintile of income brackets always turn to suffer non adherence to service utilization. This could partly due to lack means to purchase the services or ignorance. These findings are in line with other studies published elsewhere in literature [18–21].

We also saw that age was statistically significant for adherence to dietary recommendation. Increasing age (years) was shown to be negatively associated with adherence to dietary recommendation. This result could also be true because diabetes duration or age during diabetes have significant influence on adherence to therapy regimens [22]. Having DM in older age comes with many challenges. For instance, older people in general are less mobile and may have other health problems or conditions that affect how they care for themselves [23]. Again, older people with DM may also have other problems like memory loss, depression, and infections that take time to heal [24, 25]. The aged may also have trouble remembering what and when to eat meals and snacks [26]. When any or more of these conditions are present, adherence to therapy regimens among the aged could be compromised and thus affect diabetes management.

Marital status was also shown to be associated with adherence to dietary recommendation. Being married was shown to be statistically significant for adherence to dietary recommendation. These results could also be true, because ideally, marriage people offer companionship and security to each other. Therefore, when one or both couple is/are financially sound, in time of crisis they are better able to support each other than single individuals. Study conducted in South Africa indicates that family (husband or wife) support for patients with DM are main predictors for adherence to dietary recommendation [27]. Other study in Niger confirmed this finding by reporting that significant association exists between marital status and adherence to dietary recommendation [28]. During crisis situation in every family, the first point of contact is the husband or wife, therefore, having maximum support from spouse in times of need like DM could significantly improve adherence to therapy regimens as witness in our study.

We also notice statistically significant association  for social support system (families and friends support)  and adherence to dietary recommendation. Increasing moderate social support system (families and friends support) was shown to be statistically significant for adherence to dietary recommendation. Social supports are strong predictors for therapy adherence in diabetes management. Social support system provides patients with practical skills and morale to manage their condition through therapy adherence. Social supports also help patients buffer stresses that come with their illness [29]. When patients received maximum support from families and friends in time of disease, they are better able to manage these stresses. It has been reported that receiving social support from both families and friends in time of disease episodes contributes to higher adherence to therapy regimens [28]. Persons with diabetes are still part of the wider society and therefore, need approval from both families and friends to successfully navigate through society. When families and friends do not understand the importance of helping parsons with diabetes stay healthy, but stigmatized them in terms of their foods selections and consumption, these patients may not be able to adhere to their treatment protocols given to them and thus stand at increased risk of treatment non adherence. When parsons with DM are not stigmatized or discriminated by their love ones but instead given encouragement, it will help them feel secured and thus optimized adherence to dietary recommendation. Patients with DM are faced with situational obstacles when it comes to food selections and consumptions on daily basis because of fear of social disapproval [30]. Study from Singapore indicates that lack of social support from families and friends of persons with diabetes were strong barriers to adherence in dietary recommendations [31] which is consistent with our results.

Socioeconomic status was also found to be statistically significant for adherence to dietary recommendation. Moderate and high socioeconomic statuses were associated with adherence to dietary recommendation. Socioeconomic status is another strong predictor for therapy adherence in diabetes management [32]. Study in Ethiopia indicates that patients who reported low monthly income also significantly reported non-adherence to dietary recommendation [33]. Other study conducted elsewhere also indicated socioeconomic limitations as strong variable  militating against adherence to dietary recommendation [34]. This study found that persons who reported low socioeconomic status have difficulty in purchasing foods items prescribed to them in their diets plan [34]. However, the authors suggested that adherence to dietary recommendation could occur among persons with modest or moderate income status. These findings have also been seen  in our results. In our study, we found that high and moderate socioeconomic statuses were statistically significant for adherence to dietary recommendation.

 Since socioeconmic status has been repeatedly reported in studies as a strong variable in therapy non adherence, we propose that when offering dietary counseling to patients with DM, dietitians should assess patients to understand their socioeconomic  status in order to better counsel them for optimum adherence. When patients’ actual socioeconomic levels are established, dieticians can collaborate with them and design appropriate eating plan that will reflect their financially status, and thus fit well into their ability to afford which will consequently leads to optimize therapy adherence. Although we found significant associations between our study variables and adherence to dietary recommendation, we cannot conclusion that causal associations exist between our study variables and adherence to dietary recommendation. This is because of possible biases and study limitations. Some of the limitations in our study are that: We used hospital-based cross sectional survey with relatively small sample size (530) to arrive at our findings. Since the cross sectional survey and relatively small sample size could not permit us to make strong conclusions that significant causal associations exist between our study variables and adherence to dietary recommendation, we therefore recommend that future studies should consider a relatively larger sample size and more powerful study designs like cohort study, case control or clinical trial to evaluate this phenomenon. Notwithstanding the poor study design and relatively small sample size, we strongly recommend that dieticians caring patients with DM should consider their social relation with their close friends and families, and also try to establish patients’ socioeconomic status in order to giving appropriate dietary recommendation that will reflect their status for optimum adherence.

## Conclusion

Adherence to recommended dietary guidelines is an important factor in diabetes management. In this study we found that living with diabetes between 6 and 11 years, being married and received social support from families and friends were significantly associated with adherence to dietary recommendation. We therefore recommend that health care workers caring for patients with diabetes should consider patients social relation with their close families and friends, and also consider their socioeconomic background because these variables could act as risk factors for therapy regimens non-adherence.

## Data Availability

The datasets used and/or analyzed during the current study are available from the corresponding author on reasonable request.
